# Certainty of evidence on the effects of cryotherapy, surgical wound closure, and chlorhexidine on clinical and patient-centered outcomes after third molar surgery: evidence mapping of systematic reviews and meta-analyses

**DOI:** 10.4317/medoral.26788

**Published:** 2025-02-15

**Authors:** Edmundo Marques do Nascimento-Júnior, Fábio Wildson Gurgel Costa, Paulo Ricardo Martins-Filho

**Affiliations:** 1 Graduate Program in Dentistry, Federal University of Sergipe, Aracaju, SE, Brazil; 2 Investigative Pathology Laboratory, Federal University of Sergipe, Aracaju, SE, Brazil; 3 Division of Oral and Maxillofacial Surgery, Graduate Program in Dentistry, Federal University of Ceará, Fortaleza, CE, Brazil; 4 Graduate Program in Health Sciences, Federal University of Sergipe, Aracaju, SE, Brazil

## Abstract

**Background:**

Removal of third molars often leads to complications such as pain, swelling, and trismus, impacting patient quality of life. Various strategies including cryotherapy, different suture techniques, and chlorhexidine are employed to mitigate these effects. However, the effectiveness of these interventions is still debated, as clinical trials present inconsistent and contrasting results. This study aims to assess the certainty of evidence from systematic reviews and meta-analyses regarding the effects of these interventions on clinical outcomes and patient quality of life following third molar surgery.

**Material and Methods:**

This evidence mapping followed the Global Evidence Mapping Initiative and PRISMA guidelines, utilizing databases such as PubMed, Embase, Cochrane, Web of Science, and Google Scholar until February 2024. Methodological quality was assessed via AMSTAR-2 and the effects of these interventions on outcomes of interest were classified as "beneficial", "probably beneficial", "harmful", "no effect", or "inconclusive". Findings were mapped using the PyMeta platform.

**Results:**

Thirteen studies were reviewed. All systematic reviews evaluated the effects of these interventions on clinical outcomes following third molar surgery, but none assessed the impact on patient quality of life. Cryotherapy was classified as probably beneficial for reducing pain and swelling within the first 72 hours post-surgery. Secondary surgical wound closure was effective in reducing pain, swelling, and trismus during the first postoperative week, but it did not mitigate the risk of bleeding, infection, or alveolitis. Chlorhexidine, especially when used as a mouthwash, is effective in preventing postoperative alveolitis. However, most reviews (76.9%) were rated as "critically low" methodological quality.

**Conclusions:**

Although the potential benefits of cryotherapy, secondary surgical wound closure, and chlorhexidine on clinical outcomes, this study revealed a predominantly low quality of evidence from systematic reviews and meta-analyses. Moreover, further research should expand investigations into the patient-centered outcomes to better guide clinical practice.

** Key words:**Third molar, oral surgery, postoperative complications, pain, edema, cryotherapy, suture techniques, chlorhexidine, evidence-based dentistry.

## Introduction

The extraction of third molars is a common dental procedure, often indicated due to complications such as impaction or misalignment (1,2). Despite its routine nature, this procedure can lead to several postoperative complications, including pain, swelling, trismus, alveolitis, and infections (3,4), significantly affecting patient's daily activities and overall well-being (5). To mitigate these adverse outcomes, various preventive and therapeutic strategies are employed in clinical practice, such as cryotherapy (4,6), diverse wound closure techniques (7,8), and the use of antiseptics like chlorhexidine (9,10).

However, the efficacy of these interventions remains a topic of debate in the literature, with some clinical trials reporting modest or inconclusive results, while others demonstrate significant improvements in postoperative outcomes (7,11). This inconsistency underscores the need for comprehensive and systematic evaluations of the existing evidence. Systematic reviews and meta-analyses address this need by synthesizing data from multiple studies and critically assessing the certainty of available evidence, thereby providing more reliable conclusions regarding the effectiveness of these interventions.

This study aims to assess the certainty of evidence from systematic reviews and meta-analyses regarding the effects of cryotherapy, surgical wound closure, and chlorhexidine on clinical outcomes and quality of life in patients undergoing third molar surgery. Through this evidence mapping, we aim to identify and address existing gaps in the literature, thereby informing future research and optimizing outcomes following third molar extractions.

## Material and Methods

We conducted an evidence mapping according to the Global Evidence Mapping Initiative methodology (12) and adhered to the PRISMA Extension for Scoping Reviews (13) (Supplement 1). Our approach also included an assessment of the methodological quality of the reviews included (14). The protocol for our study was registered a priori on the Open Science Framework platform (https://doi.org/10.17605/OSF.IO/DAST9).

- Research question

This evidence mapping was conducted to address the following research question: “What is the certainty of the available evidence regarding the effects of cryotherapy, surgical wound closure, and chlorhexidine on clinical outcomes and quality of life after third molar surgery?”

- Eligibility criteria

We included systematic reviews regardless of publication year or language. These reviews must focus on evaluating the effectiveness of cryotherapy, surgical wound closure techniques, and the use of chlorhexidine in managing postoperative complications following third molar extractions. Each systematic review had to report at least one of the following outcomes: pain, swelling, trismus, bleeding, surgical wound infection, or alveolitis. Additionally, we sought to identify reviews that assessed the impact of these complications on the quality of life of patients undergoing these procedures. In cases of updated systematic reviews, both studies were retained, as many reviews improve their methodology and address limitations over time. Systematic reviews without meta-analysis, overview-type studies, and network meta-analyses were excluded.

- Search strategy

We conducted a systematic search in four databases (PubMed, Embase, Cochrane, and Web of Science) in September 2023, with an update in February 2024, to identify systematic reviews and meta-analyses relevant to our study. Additionally, we evaluated the top 100 search results on Google Scholar. Our search strategy used a comprehensive approach, incorporating Medical Subject Headings (MeSH) and Entry Terms (EMTREE), along with specific keywords related to our topics of interest, including variations such as: "Third molar", "Third molars", "Wisdom tooth", "Wisdom teeth", "Cryotherapy", "Cold therapy", "Ice", "Suture", "Sutures", "Suture technique", "Suture techniques", "Closure technique", "Closure techniques", "Closure ways", "Primary closure", "Secondary closure", "Wound closure", "Wound healing", "Surgical wound", and "Chlorhexidine".

To ensure thorough coverage, we tailored the search strategy for each database. Additionally, we performed a manual search through the reference lists of the included systematic reviews to identify any relevant studies might have been overlooked during the electronic database searches. We conducted separate searches for each intervention of interest—cryotherapy, surgical wound closure techniques, and chlorhexidine—to guarantee thorough and pertinent results for each intervention. Detailed search strategies for each database are outlined in the Supplement 2, providing transparency and facilitating the reproducibility of our search methodology for future research.

- Selection of systematic reviews

We managed all retrieved titles and abstracts using the Ryyan platform (available at https://www.rayyan.ai/). After removing duplicates, two reviewers (EMNJ and PRMF) independently screened all titles and abstracts for potentially relevant studies. Subsequently, potentially eligible articles were set aside for full-text reading and decision-making. Discrepancies were resolved by consensus or consultation with a third reviewer (JWGC). Detailed explanations were provided for the exclusion of any study after full-text review.

- Methodological quality assessment of systematic reviews

We evaluated the methodological quality of each included systematic review using the AMSTAR-2 tool (15). This validated instrument consists of 16 items specifically designed to critically assess the quality of systematic reviews. The overall rating is derived from identified weaknesses in key domains, notably items 2, 4, 7, 9, 11, 13, and 15. Confidence in the review outcomes is classified into four levels: "High", indicating no or only one non-critical weakness; "Moderate", indicating the presence of more than one non-critical weakness; "Low", applicable when there is one critical flaw, with or without additional non-critical weaknesses; and "Critically Low", applicable when multiple critical flaws are present, with or without non-critical weaknesses.

Two researchers (EMNJ and PRMF) independently conducted the methodological quality assessments using the tool’s online checklist (https://amstar.ca/Amstar_Checklist.php). Following the discussion of individual findings, we compiled a Table detailing the quality of evidence rating for each review. If a review was authored by one of the researchers responsible for this phase, a third reviewer (EMNJ and PRMF) was appointed for the assessment.

- Data extraction

Data extraction was also performed by two researchers (EMNJ and PRMF), independently, using a pre-formatted Excel spreadsheet. Discrepancies were resolved by consensus or consultation with a third reviewer (EMNJ and PRMF). The following information was extracted from the selected systematic reviews:

1. Authors, country, and year of publication.

2. Number of clinical trials included.

3. Population.

4. Interventions: cryotherapy, surgical wound closure, or chlorhexidine.

5. Comparisons.

a. For cryotherapy: non-use of ice.

b. Surgical wound closure: primary closure with suture versus secondary closure.

c. For chlorhexidine: placebo, any other substance used for the same purpose, or no treatment.

6. Outcomes of interest:

a. For cryotherapy: pain, swelling, trismus, and quality of life.

b. For wound closure: pain, swelling, trismus, bleeding, surgical wound infection, alveolitis, and quality of life.

c. For chlorhexidine: surgical wound infection, alveolitis, and quality of life.

7. Tool used for risk of bias assessment.

8. Assessment of evidence certainty through the Grading of Recommendations Assessment, Development, and Evaluation (GRADE) (16).

- Effects of interventions on outcomes of interest

The impacts of the interventions on the outcomes of interest were evaluated primarily through findings from meta-analyses, with a particular focus on forest plots. This analysis extended beyond mere summary measures; it also examined the direction and confidence intervals of the effects observed in individual studies included in each meta-analysis. Such a comprehensive approach enables a detailed interpretation of results, enhancing our understanding of the effect magnitude of the assessed interventions.

The effects of cryotherapy, surgical wound closure, and chlorhexidine in the postoperative period after third molar removal were classified as: 1) "Beneficial": when the summary measure demonstrated a significant reduction in postoperative complications, supported by confidence intervals from individual studies that mostly did not cross the null line, indicating a clear improvement in the outcomes of interest; 2) "Probably beneficial": when the summary measure showed a reduction in postoperative complications, although with confidence intervals from individual studies approaching the null line. Such results indicated a possible advantage of the interventions, requiring, however, more evidence for a definitive conclusion; 3) "Harmful": interventions were considered harmful when the summarized results pointed to a significant increase in postoperative complications compared to the comparison group; 4) "No effect": this classification was applied when the summary measure did not demonstrate a significant difference in postoperative complications between compared groups; 5) "Inconclusive": assigned to interventions whose results did not allow for a clear interpretation due to limitations such as wide confidence intervals, insufficient studies, or significant variability in the results of the studies. This category reflects the need for additional research to clarify the effect of the interventions.

- Presentation of evidence mappings

We displayed the evidence mappings using bubble charts, where each bubble corresponds to a systematic review. These charts provide information in three dimensions: 1) the classification of the effects of the interventions represented on the X-axis; 2) the AMSTAR-2 assessment on the Y-axis; and 3) the author, year, and size of the population included in each systematic review above each bubble, with the size of the bubble proportional to the size of the population. Additionally, we also presented Tables describing the characteristics of the studies shown in the bubble charts, including information about authors, follow-up period, measure, and effect. The Figures were generated using the PyMeta platform (https://www.pymeta.com/evdmap/).

## Results

- Selected studies

**Figure 1:**
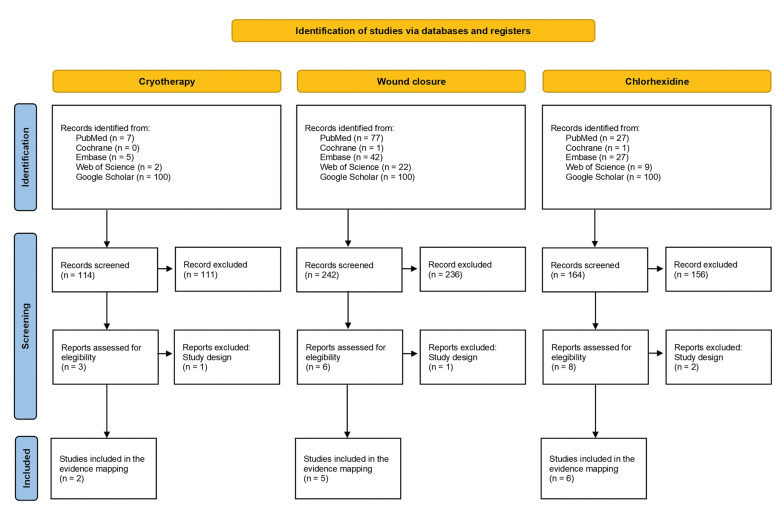
PRISMA flowchart detailing the selection process.

The database search resulted in 520 records, including 114 for cryotherapy, 242 for surgical wound closure methods, and 164 for chlorhexidine. Following the initial screening based on titles and abstracts, 17 studies were selected for full-text review (three related to cryotherapy, six to wound closure, and eight to chlorhexidine. Out of these, 13 systematic reviews (two on cryotherapy, five on wound closure, and six on chlorhexidine) (17-29) met the eligibility criteria. The search and selection process are thoroughly detailed in Fig. 1 list of excluded studies, with the reasons for their exclusion, is provided in the Supplement 3.

- Characteristics of included systematic reviews

All included systematic reviews conducted meta-analyses for at least one of the evaluated outcomes and were published in English from 2005 to 2024. Two reviews (17,18) specifically focused on the effects of cryotherapy, four (19,21-23) exclusively on surgical wound closure methods, and five (24-27,29) on the use of chlorhexidine. One review (28) examined various interventions for alveolitis, though data pertaining only to chlorhexidine were extracted for this study. Another review (20) investigated multiple interventions and their impacts on various outcomes; however, we extracted data related to wound closure.

All studies investigating cryotherapy evaluated edema and trismus, but only one (18) assessed pain. In the case of surgical wound closure, all studies assessed pain, edema, and trismus; four studies additionally explored bleeding (19-21,23), and three surgical wound infection and alveolitis (19-21). As for the studies on chlorhexidine, all systematically reviewed alveolitis. None of the studies across these interventions reported data on quality of life.

The number of clinical trials included in each review varied from 4 to 40. Twelve reviews used a tool to assess the quality of primary studies, with eleven using the Cochrane Collaboration tool (17-23,26-29) and one using the Macaskill, Gleser-Olkin, and Rosenthal method (25) Only six reviews applied the GRADE methodology (18-21,23,28) to assess the quality of evidence from individual studies. Table 1 presents the general characteristics of the included systematic reviews.

- Methodological quality of systematic reviews

**Figure 2:**
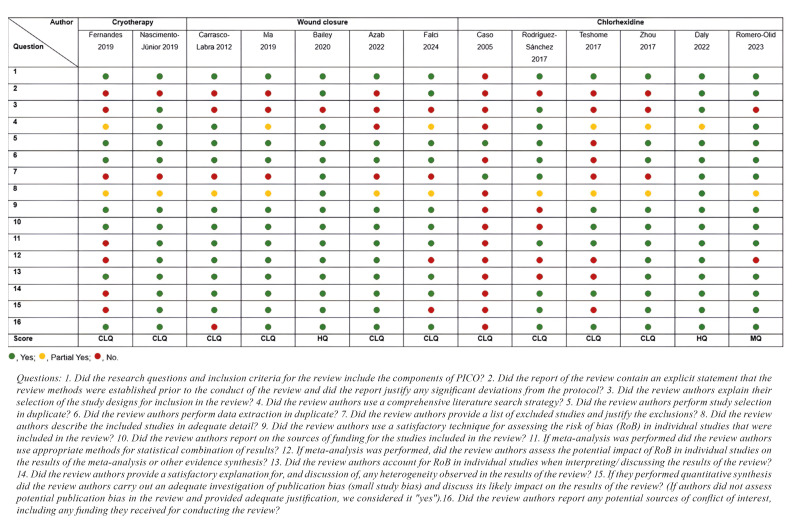
Assessment of the Methodological Quality of Systematic Reviews (AMSTAR-2) included.

Ten systematic reviews (17-19,21-27) were rated as "critically low", one (29) as "moderate", and only two (20,28) as "high" methodological quality, according to the AMSTAR-2 criteria. Most reviews were downgraded for not referencing a protocol (nine studies; 69.2%) and for not providing a list of excluded studies (eight studies; 61.5%). All information regarding the application of AMSTAR-2 is detailed in Fig. 2.

- Evidence mapping and effects of interventions of interest

The evidence mapping on cryotherapy, surgical wound closure type, and the use of chlorhexidine in reducing pain, swelling, and trismus, and in preventing bleeding, infection, and alveolitis after third molar removal surgery revealed 66 analyses on the effects of these interventions across various controls and outcomes.

- Cryotherapy

**Figure 3:**
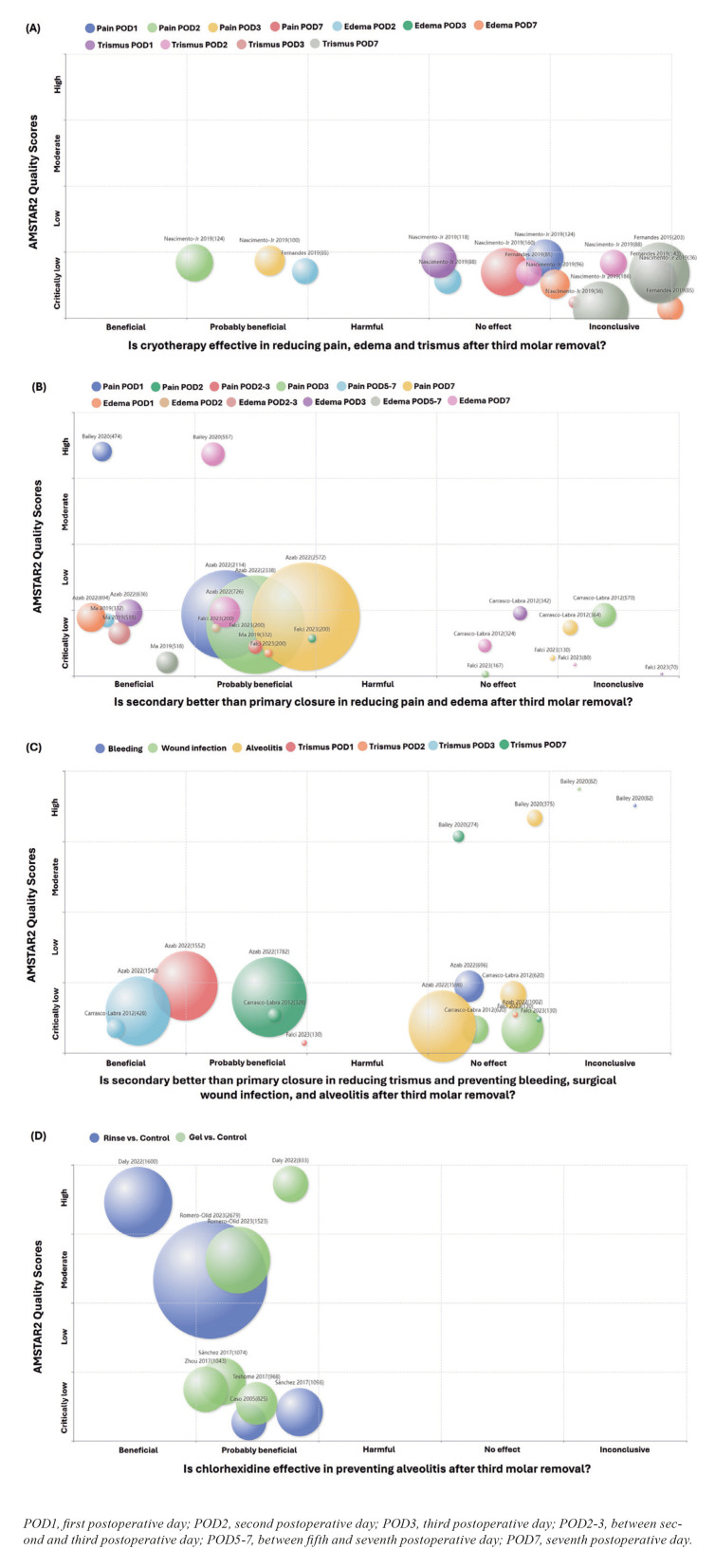
Evidence mappings on the effects of (A) cryotherapy, (B and C) surgical wound closure, and (D) chlorhexidine on clinical outcomes after third molar surgery.

Two systematic reviews (17,18) evaluated the effectiveness of cryotherapy in reducing pain, swelling, and trismus. Cryotherapy was deemed "probably beneficial" for reducing pain on the second and third postoperative days, as well as for diminishing swelling on the second postoperative day. However, the quality of the studies that provided these findings was rated as "critically low" according to AMSTAR-2 criteria. There is no evidence to support the effectiveness of cryotherapy in alleviating pain on the first- and seventh-days post-surgery, reducing swelling from the third postoperative day, or in attenuating trismus at any later stage following the surgical procedure. All information extracted regarding the effects of cryotherapy is detailed in Table 2 and a bubble chart displaying these results is available in Fig. 3.

- Surgical wound closure

Five systematic reviews (19-23) evaluated the efficacy of secondary closure, compared to primary closure, in reducing pain, swelling, and trismus, and in preventing bleeding, wound infection, and alveolitis. Generally, secondary closure was considered "beneficial" or "probably beneficial" in reducing pain, swelling, and trismus during the first week of postoperative. However, the evidence quality was considered "high" only for reducing pain on the first postoperative day and swelling after seven days of surgery. For other outcomes and evaluation times, the evidence quality was "critically low". No evidence was found supporting the reduction of bleeding, infection, or alveolitis risk with the secondary closure technique. All information extracted about the effects of secondary surgical wound closure is presented in Table 3 and bubbles charts with these results can be seen in Fig. 3.

- Chlorhexidine

Six systematic reviews assessed the efficacy of chlorhexidine as a preventive agent for postoperative alveolitis (24-29). The best available evidence characterizes chlorhexidine, especially when used as a mouthwash, as "beneficial" or "probably beneficial" in preventing alveolitis. All information extracted for the effects of chlorhexidine is recorded in Table 4 and a bubble chart with these results can be seen in Fig. 3.

## Discussion

This study represents one of the initial efforts in oral surgery to employ evidence mapping for third molar removal, assessing the certainty of evidence concerning the effects of cryotherapy, surgical wound closure, and chlorhexidine in alleviating common postoperative complications. While not intended to supplant established clinical protocols or guidelines, our analysis provides a thorough and critical synthesis that highlights both the potential advantages and constraints of these interventions. Recommendations arising from our findings should be interpreted within the specific clinical context of each patient, considering alternative approaches, cost-effectiveness, and other pertinent contextual factors.

The results of our evidence mapping indicate a limited and variable quality among systematic reviews assessing the discussed interventions and outcomes. From a total of 520 records found across multiple databases, only 17 systematic reviews were identified and selected for full reading. Of these, only 13 met the inclusion criteria. Although most of the included reviews present a limited number of primary studies, all of them were based on clinical trial data, which is considered the highest level of evidence for evaluating the efficacy of therapeutic interventions (30).

However, the methodological quality of the reviews analyzed varied significantly, with many categorized as "low" or "critically low". This study underscores the prevalence of methodological shortcomings, including the lack of predefined protocols, insufficient justification for including certain study types, and a lack of publication bias assessment. Such deficiencies can undermine the reliability of the results and, consequently, the credibility of clinical recommendations derived from these reviews. Although not a required step in evidence mapping, it is recommended that all types of reviews incorporate a methodological quality assessment to ensure the consistency of their conclusions (14,31).

Despite the essential role of the AMSTAR-2 tool in evaluating the methodological quality of systematic reviews, we recognize its limitations. The AMSTAR-2 scoring system emphasizes critical items, specifically questions 2, 4, 7, 9, 11, 13, and 15, where a single critical flaw can downgrade an otherwise "high-quality review to "low" or "critically low". This approach can result in assessments that do not accurately reflect the robustness of a review. For instance, reviews with multiple minor issues might receive a more favorable rating than those with a single critical error. Moreover, AMSTAR-2 tends to overemphasize the procedural components of conducting a review, potentially neglecting other crucial elements such as the clinical relevance of the research questions and the accurate interpretation of findings.

The tool also contains ambiguities in some of its critical questions, which may lead to assessment inconsistencies among different reviewers. is the question regarding the analysis of publication bias, which is deemed a critical failure if not conducted, even in scenarios where such analysis may not be applicable due to a limited number of studies (32). This strict adherence to criteria can unjustly penalize reviews that, for methodologically sound reasons, do not perform this analysis. Thus, it is essential for users of AMSTAR-2 to interpret the ratings with caution, acknowledging these limitations and opting for a more comprehensive evaluation of the methodological quality of systematic reviews. Recognizing these constraints is crucial for the effective use of the tool, ensuring that conclusions drawn from its application are both reliable and relevant for clinical practice and health policy development.

Systematic review results indicate that immediate post-surgery cryotherapy effectively reduces pain and swelling, supporting its recognized anti-inflammatory and analgesic effects. The pain reduction achieved through cryotherapy primarily results from the blockade of nerve impulse transmission when local temperatures drop below 14°C, leading to pain perception reduction (17). This effect is influenced by variables such as the size and shape of the ice pack, application duration and frequency, tissue thickness at the application site, and methodological differences across studies (18). Moreover, cooling contributes to reduced postoperative swelling through its vasoconstrictive effects, which decrease neutrophil activity and inflammatory cytokine release, thus mitigating fluid accumulation (17). However, the results of reducing trismus are inconsistent, suggesting a need for more standardized cryotherapy protocols and further research into its optimal application parameters.

Traditional suturing techniques following third molar extractions aim to promote primary wound closure and minimize risks of postoperative bleeding and infection (33). However, emerging evidence suggests that secondary wound closure, which involves leaving the wound partially open, may more effectively alleviate pain and swelling post-surgery. This method promotes the interaction between the alveolus and the oral cavity, facilitating better drainage of inflammation (34). In contrast, suturing can induce trauma, damage capillaries, and trigger pro-inflammatory cytokines release, increasing vascular permeability and consequently swelling (35). Despite these potential benefits, the data regarding secondary closure's effectiveness in reducing trismus, bleeding, and infection risks remain inconsistent and limited, underscoring the need for additional research to establish the efficacy of secondary closure across various clinical outcomes.

In recent years, chlorhexidine has become increasingly popular as an antiseptic in medicine and dentistry, favored for its antimicrobial efficacy, low cost, and ease of application (36). Particularly when used as a mouthwash, the best evidence indicates that chlorhexidine is effective in reducing postoperative alveolitis, while its application in gel form is considered probably beneficial in preventing this complication. The effectiveness of chlorhexidine is derived from its ability to disrupt biofilms and diminish oral pathogen loads (37), attribuTable to its broad-spectrum antimicrobial activity against both gram-positive and gram-negative bacteria. It functions as a bacteriostatic agent at lower doses and exhibits bactericidal properties at higher concentrations, with additional antifungal effects (38).

It is noteworthy that none of the systematic reviews included in this study assessed the impact of interventions on patients' quality of life, a key outcome for patient-centered evaluations. The omission of this critical dimension is concerning, as comprehending patients’ quality of life, satisfaction, and overall well-being is essential for informed decision-making and tailoring treatments to optimize patient experiences during third molar surgeries (1). This research gap significantly hinders our understanding and limits our ability to provide personalized care that aligns with individual patient needs. Prioritizing the inclusion of quality of life measures in future research should be a focus, ensuring these metrics are standard outcomes in clinical studies concerning third molar removals.

The primary limitation of this evidence mapping lies in its inability to analyze clinical and methodological heterogeneities among the trials included in the meta-analyses reviewed. These heterogeneities are critical for interpreting results, as they include differences in the characteristics of the third molars removed, variations in surgical procedures performed, the experience levels of the surgeons involved, and the use of medications in the pre- and post-operative phases. Such variations can significantly impact clinical outcomes and the efficacy of the interventions studied. The inability to detail and adjust for these clinical and methodological differences restricts the generalizability of the findings and may compromise the applicability of the clinical recommendations derived from this study. This issue underscores the need for more detailed and specific analyses that consider these critical variables in the planning of future research and in the formulation of evidence-based guidelines.

## Conclusions

This evidence mapping revealed a predominantly low methodological quality among systematic reviews and meta-analyses that evaluated the effects of cryotherapy, surgical wound closure, and chlorhexidine following third molar surgery. Regarding the interventions:

1. Cryotherapy showed probable benefits in reducing pain and swelling on the second postoperative day. However, its effectiveness on other postoperative days and for reducing trismus remains unsupported by high-quality evidence. These findings suggest that while cryotherapy may be beneficial shortly after surgery, its broader applications require more robust investigation.

2. Secondary closure was generally found to be beneficial or probably beneficial in reducing pain, swelling, and trismus during the first week post-surgery. However, high-quality evidence supports its benefit only in reducing pain on the first postoperative day and swelling after seven days. There is no substantial evidence supporting its effectiveness in reducing bleeding, infection, or alveolitis risk, highlighting the need for more high-quality research in these areas.

3. Chlorhexidine, particularly as a mouthwash, was consistently beneficial in preventing postoperative alveolitis. This suggests that chlorhexidine remains a valuable antiseptic tool in managing complications following third molar extractions.

In conclusion, while some interventions show potential benefits, the overall low quality of the evidence necessitates cautious interpretation of these results. Future research should prioritize improving methodological standards and expanding investigations into the patient-centered outcomes and the longitudinal effects of these interventions to better guide clinical practice.

## Figures and Tables

**Table 1:** Characteristics of the meta-analyses included in the evidence mapping.

	Author (year)	Trials	Intervention vs. Control		Outcomes of interest
			Comparison	Time	
Cryotherapy	Fernandes (2019)	4	Ice pack vs. no use of ice	Immediately and for 24h post-surgery	Edema and trismus
	Nascimento-Júnior (2019)	6	Ice pack vs. no use of ice	Immediately and for 24h post-surgery	Pain, edema, and trismus
Wound closure	Carrasco-Labra (2012)	14	Secondary vs. primary closure	Immediately after surgery	Pain, edema, trismus, bleeding, wound infection, and alveolitis
	Ma (2019)	5	Secondary vs. primary closure	Immediately after surgery	Pain, edema, and trismus
	Bailey (2020)	8	Secondary vs. primary closure	Immediately after surgery	Pain, edema, trismus, bleeding, wound infection, and alveolitis
	Azab (2022)	40	Secondary vs. primary closure	Immediately after surgery	Pain, edema, trismus, bleeding, wound infection, and alveolitis
	Falci (2024)	7	Secondary vs. primary closure	Immediately after surgery	Pain, edema, trismus, and bleeding
Chlorhexidine	Caso (2005)	7	CHX rinse vs. placebo or others*	Pre- and post-surgery	Alveolitis
	Rodríguez-Sánchez (2017)	18	CHX rinse and gel vs. placebo or others*	Rinse: pre- and post-surgery. Gel: intra-socket	Alveolitis
	Zhou (2017)	11	CHX gel vs. placebo or others*	Not specified	Alveolitis
	Teshome (2017)	10	CHX gel vs. placebo or others*	Not specified	Alveolitis
	Daly (2022)	15	CHX rinse and gel vs. placebo or others*	Rinse: before and 24h post-surgery. Gel: intra-socket	Alveolitis
	Romero-Olid (2023)	33	CHX rinse and gel vs. placebo or others*	Not specified	Alveolitis

CHX, chlorhexidine. *Others: Saline solution, dressings, or no treatment.

**Table 2:** Synthesis of extracted data on cryotherapy for reduction of pain, edema, and trismus after third molar extraction.

	Author (year)	Sample Size	Follow-up	Measurement	Effect
Pain	Nascimento-Junior (2019)	124	POD1	MD 0.17 (-0.67 to 1.01)	No effect
	Nascimento-Junior (2019)	124	POD2	MD -0.72 (-1.45 to -0.01)	Probably beneficial
	Nascimento-Junior (2019)	100	POD3	MD -0.36 (-0.59 to -0.13)	Probably beneficial
	Nascimento-Junior (2019)	160	POD7	MD -0.46 (-1.28 to 0.37)	No effect
Edema	Nascimento-Junior (2019)	88	POD2	SMD -0.28 (-0.88 to 0.31)	No effect
	Nascimento-Junior (2019)	36	POD3	SMD -2.26 (-3.12 to -1.40)	Inconclusive
	Nascimento-Junior (2019)	96	POD7	SMD -0.52 (-1.92 to 0.89)	Inconclusive
	Fernandes (2019)	85	POD2	MD -0.94 (-1.49 to -0.39)	Probably beneficial
	Fernandes (2019)	85	POD7	MD -0.35 (-1.34 to 0.64)	Inconclusive
Trismus	Nascimento-Junior (2019)	118	POD1	MD -0.27 (-3.81 to 3.26)	No effect
	Nascimento-Junior (2019)	88	POD2	MD 0.22 (-3.49 to 3.93)	Inconclusive
	Nascimento-Junior (2019)	36	POD3	MD -0.63 (-1.60 to 0.34)	Inconclusive
	Nascimento-Junior (2019)	186	POD7	MD -0.04 (-0.63 to 0.54)	Inconclusive
	Fernandes (2019)	143	POD1	MD 0.55 (-2.14 to 3.24)	Inconclusive
	Fernandes (2019)	85	POD2	MD 2.37 (-0.50 to 5.24)	No effect
	Fernandes (2019)	203	POD7	MD 0.76 (-3.10 to 4.63)	Inconclusive

POD1, first postoperative day; POD2, second postoperative day; POD3, third postoperative day; POD7, seventh postoperative day; MD, mean difference; SMD, standardized mean difference. Negative effect measures for continuous outcomes indicated favorability towards cryotherapy.

**Table 3:** Synthesis of extracted data on secondary versus primary closure for reduction of pain, edema, and trismus, and prevention of bleeding, surgical wound infection, and alveolitis after third molar removal.

	Author (year)	Sample Size	Follow-up	Measurement	Effect
Pain	Carrasco-Labra (2012)	570	POD3	SMD -0.29 (-0.63 to 0.05)	Inconclusive
	Carrasco-Labra (2012)	364	POD7	SMD 0.00 (-0.19 to 0.19)	Inconclusive
	Ma (2019)	332	POD2-3	SMD -0.49 (-0.71 to -0.27)	Probably beneficial
	Ma (2019)	332	POD5-7	SMD -1.12 (-1.57 to -0.66)	Beneficial
	Bailey (2020)	474	POD1	MD -0.94 (-1.38 to -0.50)	Beneficial
	Azab (2022)	2114	POD1	MD -1.12 (-1.46 to -0.78)	Probably beneficial
	Azab (2022)	2338	POD3	MD -0.97 (-1.26 to -0.69)	Probably beneficial
	Azab (2022)	2572	POD7	MD -0.30 (-0.41 to -0.19)	Probably beneficial
	Falci (2024)	200	POD1	MD -1.08 (-1.35 to -0.81)	Probably beneficial
	Falci (2024)	200	POD2	MD -0.50 (-0.83 to -0.17)	Probably beneficial
	Falci (2024)	167	POD3	MD -0.05 (-0.75 to 0.66)	No effect
	Falci (2024)	130	POD7	MD 0.00 (-0.03 to 0.03)	No effect
Edema	Carrasco-Labra (2012)	342	POD3	SMD -0.37 (-0.76 to 0.02)	No effect
	Carrasco-Labra (2012)	324	POD7	SMD -0.15 (-0.39 to 0.10)	No effect
	Ma (2019)	518	POD2-3	SMD -0.36 (-0.54 to -0.19)	Beneficial
	Ma (2019)	518	POD5-7	SMD -0.51 (-0.68 to -0.33)	Beneficial
	Bailey (2020)	557	POD7	MD -0.33 (-0.57 to -0.09)	Probably beneficial
	Azab (2022)	694	POD1	SMD -1.07 (-1.49 to -0.65)	Beneficial
	Azab (2022)	636	POD3	SMD -1.14 (-1.75 to -0.54)	Beneficial
	Azab (2022)	726	POD7	SMD -1.11 (-1.77 to -0.45)	Probably beneficial
	Falci (2024)	200	POD1	SMD -1.23 (-2.34 to -0.11)	Probably beneficial
	Falci (2024)	200	POD2	SMD -0.66 (-1.16 to -0.16)	Probably beneficial
	Falci (2024)	70	POD3	SMD -0.14 (-0.61 to 0.33)	Inconclusive
	Falci (2024)	80	POD7	SMD 0.00 (-0.44 to 0.44)	Inconclusive
Trismus	Carrasco-Labra (2012)	428	POD3	MD -3.72 (-6.03 to -1.42)	Beneficial
	Carrasco-Labra (2012)	328	POD7	MD -2.35 (-4.33 to -0.37)	Probably beneficial
	Bailey (2020)	274	POD7	MD 0.29 (-0.32 to 0.90)	No effect
	Azab (2022)	1552	POD1	MD -4.25 (-5.70 to -2.79)	Beneficial
	Azab (2022)	1540	POD3	MD -4.14 (-5.84 to -2.45)	Beneficial
	Azab (2022)	1782	POD7	MD -2.58 (-3.75 to -1.41)	Probably beneficial
	Falci (2024)	130	POD1	SMD -1.04 (-1.41 to -0.67)	Probably beneficial
	Falci (2024)	130	POD2	SMD -1.54 (-3.31 to 0.23)	No effect
	Falci (2024)	130	POD7	SMD -1.26 (-3.14 to 0.62)	No effect
Bleeding	Bailey (2020)	82	30 days	RR 2.45 (0.68 to 8.82)	Inconclusive
	Azab (2022)	696	2-31 days	RD 0.00 (-0.04 to 0.04)	No effect
Wound infection	Carrasco-Labra (2012)	620	1 week	RR 0.51 (0.18 to 1.47)	No effect
	Bailey (2020)	82	1 week	RR 0.21 (0.01 to 4.24)	Inconclusive
	Azab (2022)	1002	2-31 days	RD 0.01 (-0.02 to 0.03)	No effect
Alveolitis	Carrasco-Labra (2012)	620	1 week	RR 0.51 (0.18 to 1.47)	No effect
	Bailey (2020)	375	1 week	RR 1.01 (0.42 to 2.44)	No effect
	Azab (2022)	1598	20-31 days	RD 0.01 (-0.02 to 0.04)	No effect

POD1, first postoperative day; POD2, second postoperative day; POD3, third postoperative day; POD7, seventh postoperative day; POD2-3, between the second and third postoperative days; POD5-7, between the fifth and seventh postoperative days; MD, mean difference; SMD, standardized mean difference; RR, relative risk; RD, risk difference. Negative effect measures for continuous outcomes indicated favorability towards secondary closure.

**Table 4:** Synthesis of extracted data on chlorhexidine for prevention of alveolitis after third molar extraction.

	Author (year)	Formulations	Sample Size	Measurement	Effect
Alveolite	Caso (2005)	Rinse vs. Control	825	RR 0.53 (0.41 to 0.69)	Probably beneficial
	Rodríguez-Sánchez (2017)	Rinse vs. Control	1096	RR 0.58 (0.47 to 0.71)	Probably beneficial
	Rodríguez-Sánchez (2017)	Gel vs. Control	1074	RR 0.47 (0.37 to 0.60)	Probably beneficial
	Teshome (2017)	Gel vs. Control	968	RR 0.43 (0.32 to 0.58)	Probably beneficial
	Zhou (2017)	Gel vs. Control	1043	OR 0.40 (0.28 to 0.55)	Probably beneficial
	Daly (2022)	Rinse vs. Control	1600	OR 0.38 (0.25 to 0.58)	Beneficial
	Daly (2022)	Gel vs. Control	833	OR 0.44 (0.27 to 0.71)	Probably beneficial
	Romero-Olid (2023)	Gel vs. Control	1523	RR 0.40 (0.31 to 0.51)	Probably beneficial
	Romero-Olid (2023)	Rinse vs. Control	2679	RR 0.50 (0.41 to 0.62)	Probably beneficial

RR, relative risk; OR, *odds ratio*.
